# Laparoscopic anterior gastropexy for chronic recurrent gastric volvulus: a case report

**DOI:** 10.1186/1752-1947-2-244

**Published:** 2008-07-24

**Authors:** Umberto Morelli, Maurizio Bravetti, Paolo Ronca, Roberto Cirocchi, Angelo De Sol, Alessandro Spizzirri, Giammario Giustozzi, Francesco Sciannameo

**Affiliations:** 1 Università degli Studi di Perugia, Clinica Chirurgica Generale e d'Urgenza, Azienda Ospedaliera 'S Maria' Terni, Italy

## Abstract

**Introduction:**

Gastric volvulus is an uncommon clinical entity, first described by Berti in 1866. It is a rotation of all or part of the stomach through more than 180°. This rotation can occur on the longitudinal (organo-axial) or transverse (mesentero-axial) axis. This condition can lead to a closed-loop obstruction or strangulation. Traditional surgical therapy for gastric volvulus is based on an open approach. Here we report the case of a patient with chronic intermittent gastric volvulus who underwent a successful laparoscopic treatment.

**Case presentation:**

A 34-year-old woman presented with multiple episodes of recurrent upper abdominal pain associated with retching and vomiting, treated unsuccessfully with intramuscular metoclopramide. Endoscopic examination of the upper digestive tract showed a suspected rotation of the stomach, and a chronic recurrent gastric volvulus was revealed by barium meal. The patient was operated on successfully, with an anterior laparoscopic gastropexy performed as the first surgical approach.

**Conclusion:**

Experience with laparoscopic anterior gastropexy is limited only to a few described cases. Our patient was clinically and radiologically followed-up for 2 years with no evidence of recurrence, either radiological or symptomatic. Based on this result, laparoscopic gastropexy can be seen and considered as an initial 'gold standard' for the treatment of gastric volvulus.

## Introduction

Gastric volvulus (from the Latin *volvere *meaning 'to roll') is an uncommon clinical entity, first described by Berti in 1866 [1]. It is a rotation of all or part of the stomach through more than 180°. This rotation can occur on its longitudinal (organo-axial) or transverse (mesentero-axial) axis. This condition can lead to a closed-loop obstruction or strangulation. It is seen both in children and elderly patients, but the majority of cases are observed in the fifth decade of life. In almost 75% of cases the volvulus is secondary to other causes (para-oesophageal hiatus hernia, traumatic diaphragmatic hernia, eventration of the diaphragm, abdominal bands or adhesions) [2]. It can be classified also as sub-diaphragmatic or primary volvulus, which is not associated with diaphragmatic disorders, or supradiaphragmatic or secondary volvulus, which is associated with diaphragmatic pathologies. Traditional surgical therapy for gastric volvulus is based on an open approach, but the use of a laparoscopic technique is now advocated for multiple pathologies that once were treated with traditional surgery.

Here we report the case of a patient with chronic intermittent gastric volvulus who underwent a successful laparoscopic treatment.

## Case presentation

A 34-year-old woman presented with multiple episodes of recurrent upper abdominal pain associated with retching and vomiting. All episodes were treated at home with intramuscular metoclopramide, not requiring hospitalization. Her clinical examination noted only mild tenderness on upper abdominal examination. Haematological and biochemical profiling was performed, as were plain abdominal X-ray and abdominal sonography, and all were normal. She also underwent an endoscopic examination of the upper digestive tract, which revealed a suspected stomach rotation. Following this, the patient ingested a barium meal that showed an organo-axial rotation of this organ, confirming the presence of a volvulus. The patient was treated with nasogastric drainage, intravenous fluids and proton pump inhibitors. She was offered definitive surgical treatment for her condition by laparoscopic gastropexy.

Laparoscopy was performed under general endotracheal anaesthesia. A carboperitoneum of 12 to 14 mmHg was created through umbilical Veress needle insertion. A number 10 trocar was placed here for camera passage. A number 10 trocar was inserted in the right iliac fossa and a number 5 trocar was inserted in the left iliac fossa. A great number of peritoneal adhesions were found on examination of the abdominal cavity. No macroscopic diaphragmatic defect was found. A laxity of gastrocolic and gastrophrenic ligaments was found, associated with a medium-grade gastrectasis.

The major curve was approached by making an opening into the gastrocolic omentum, which was divided from the antrum to the fundus and skeletonised using an ultracision harmonic scalpel. Four Ethibond 2/0 seromuscular sutures were later placed, using the trocar ports to introduce the strands. The sutures were placed on the anterior wall of the stomach (two near the fundus on the major and lesser curve side and two on the gastric body on the major and lesser curve side). The needles were cut off, retrieved and both ends of the suture were exteriorized from the abdominal wall. The carboperitoneum was reduced by 5 mmHg in order to progress the stomach to the abdominal wall. The sutures were then tied into the subcutaneous tissue through port-site incisions. Adequate positioning of the stomach to the abdominal wall was confirmed by visualization from within. The trocars were removed and the wounds were closed.

The patient was allowed a fluid and light diet intake from the first postoperative day and was discharged on the second postoperative day. A year later she underwent a barium meal investigation which revealed no radiological abnormalities and she remained asymptomatic at a follow-up of 2 years.

## Discussion

Gastric volvulus is defined as an abnormal rotation of the stomach of more than 180° [3], creating a closed-loop obstruction, resulting finally in incarceration and strangulation. This uncommon clinical entity was first described by Berti in 1866 [1] who described an autoptical case observed in a 61-year-old woman, but it remains a rare finding in common clinical practice. In 1904 Borchardt described the classic triad of severe epigastric pain, retching with vomiting and inability to pass a nasogastric tube [4]. Gastric volvulus can be classified into three forms, organo-axial, mesenterico-axial and combined. In the first form the stomach rotates around an axis that connects the gastro-oesophageal junction and the pylorus, the antrum rotating in the opposite direction to the fundus of the stomach. The second form is characterized by the rotation around an axis that bisects both the lesser and greater curve; the rotation is usually incomplete and occurs intermittently, with uncommon vascular compromise. The combined form is rare; in this case the stomach twists both mesenterico-axially and organo-axially. This form is usually observed in patients with chronic volvulus. Various ligamentous structures normally keep the stomach in place: the gastrophrenic ligament, the gastrocolic ligament, the gastrosplenic ligament and peritoneal fixation of the duodenum. The absence or loosening of gastrocolic and gastrosplenic ligaments were demonstrated by Dalgaard to cause gastric volvulus [5].

Congenital diaphragmatic hernia, para-oesophageal hernia or wandering spleen are the main secondary causes of this condition [6-8]. Both chronic recurrent and acute gastric volvulus have been reported. Clinical findings appear to be related to the degree of rotation and subsequent gastric obstruction. They include recurrent abdominal pain, vomiting and gastric distension in the chronic recurrent form, through to clinical evidence of acute abdomen due to vascular compromising in the acute form, or as a complication of the chronic recurrent form. The Borchard triad [4] was not seen in our case as a nasogastric tube was placed without problem. Most cases are treated routinely as gastritis, with subsequent therapy based on proton pump inhibitors and/or antacids.

The diagnosis of chronic volvulus can be obtained with a barium study, showing the stomach lying horizontal and upside down, or by performing a computed tomography scan which demonstrates two bubbles with a transition line. A gastric volvulus requires treatment either in its acute presentation as an abdominal emergency or when the chronic variety becomes symptomatic, in order to prevent complications [9].

In 1968 Tanner [3] described various methods of surgical repair for gastric volvulus. These included gastrojejunostomy, fundo-antral gastrogastrostomy (Opolzer's operation), partial gastrectomy, division of bands, repair of diaphragmatic hernia, simple gastropexy, gastropexy with division of the gastrocolic omentum (Tanner's operation) and repair of eventration of the diaphragm. Most of these have become obsolete, substituted by less-invasive techniques. Endoscopic derotation of the stomach has given satisfactory results [10,11]. Given the recurrent nature of this clinical condition, endoscopic derotation can be considered as a temporary solution. In patients with high-risk pre-operative conditions, endoscopic derotation with a single or dual PEG (percutaneous endoscopic gastrostomy) tube placement has been reported to have success [12,13]. However, some concern about this procedure comes from reported gastric rotations initiated by PEG tubes [14]. There have been few reports of laparoscopic gastropexy for management of acute and chronic gastric volvulus [9,15,16]. Laparoscopy has the advantage of placing the stomach in a three-dimensional plane, giving correct sight of suture placement, with no risk to the peritoneal wall. Our technique included some important steps to prevent recurrence. The gastrocolic ligament division from antrum to fundus, as described by Tanner [3], reduced the upward pulling force and drag on the greater curvature. The use of an ultracision harmonic scalpel enabled us to achieve a precise dissection with minimal blood loss. We secured the stomach with four sutures tied to the abdominal wall through port incisions, and these transabdominal sutures provided a more secure anchorage for the stomach, to prevent recurrences.

## Conclusion

Chronic gastric volvulus is an uncommon cause of recurrent abdominal pain. Its diagnosis must be suspected in patients where no real organic aetiology can be found. Experience with laparoscopic anterior gastropexy is limited to only a few described cases. Our patient was clinically and radiologically followed-up for 2 years with no evidence of recurrence, either radiological or symptomatic. Several other authors have approached this disease in a similar manner, achieving similarly good results. Although laparoscopic gastropexy is not yet defined as the 'gold standard' for treating recurrent gastric volvulus, these results can be viewed optimistically.

## Abbreviations

PEG: percutaneous endoscopic gastrostomy.

## Competing interests

The authors declare that they have no competing interests.

## Authors' contributions

UM conceived of the study, collected data and drafted the manuscript. MB, PR, RC, ADS, AS, GG and FS helped to draft the manuscript and critically reviewed it. All authors read and approved the final manuscript.

## Consent

Written informed consent was obtained from the patient for publication of this case report and accompanying images. A copy of the written consent is available for review by the Editor-in-Chief of this journal.
